# The diagnostic utility of genetic testing in inherited thrombocytopenia: regional multicenter tertiary experience

**DOI:** 10.1016/j.rpth.2025.102869

**Published:** 2025-04-24

**Authors:** Eman Hassan, Carl Fratter, Will Lester, Charles Percy, Walaa Saad, Afrah Alkhedir, Jayashree Motwani, Patricia Bignell, Phillip L.R. Nicolson, Neil V. Morgan, Sandeep Potluri, Gillian Lowe

**Affiliations:** 1 Department of Cardiovascular Sciences, College of Medicine and Health, University of Birmingham, UK; 2 Department of Haematology, Queen Elizabeth Hospital, Birmingham, UK; 3 Oxford Centre for Genomic Medicine, Oxford University Hospitals NHS Foundation Trust, Oxford, UK; 4 Department of Haematology, Birmingham Children’s Hospital, Birmingham, UK; 5 Cancer and Genomic Sciences, College of Medicine and Health, University of Birmingham, Birmingham, UK

Thrombocytopenia is a common hematological finding caused by a heterogeneous group of conditions that can be either congenital or acquired [1]. Immune thrombocytopenia (ITP) remains a diagnosis of exclusion, which can make differentiation from heritable thrombocytopenia (HT) challenging [2]. HTs are an expanding group of genetic disorders, often with extra hematological manifestations, and a significant proportion have an associated risk of myelodysplasia or malignancy [3].

Recent advancements in genetic testing have transformed the diagnosis of inherited bleeding disorders, many of which were historically underdiagnosed or misclassified. The Bleeding and Platelet Disorders Gene Panel (R90 panel) was introduced in the National Health Service in the United Kingdom in 2020 [4]. The panel uses next-generation sequencing, specifically whole exome sequencing, with a targeted analysis of a curated panel comprising 108 genes. Detected variants are classified as pathogenic, likely pathogenic, or variants of uncertain significance following the American College of Medical Genetics and Genomics/Association for Molecular Pathology guidelines [5]. Variants of uncertain significance are included in the interpretive report when additional evidence, such as functional studies, family-based segregation, or clinical phenotype, could support their contribution to thrombocytopenia. Results are reviewed at a regional genomic laboratory hub multidisciplinary team meeting, which includes clinical and scientific representatives.

In the West Midlands, Birmingham, United Kingdom, this panel is offered to patients with unexplained chronic thrombocytopenia (>1 year, platelet count < 150 × 10^9^/L) who have no prior record of normal platelet counts, especially if they meet at least one of the following criteria: a family history of thrombocytopenia, thrombocytopenia unresponsive to standard treatments for acquired thrombocytopenia (eg, ITP), bleeding symptoms unexplained by platelet count, abnormal platelet morphology, abnormal platelet function tests, or associated physical abnormalities.

We conducted an audit of both adult and pediatric patients at comprehensive care centers in the West Midlands. Our aim was to reevaluate cases of chronic thrombocytopenia and assess the role of genetics in identifying underlying HTs. The study was approved as a multicenter service evaluation by the respective audit departments. Data were collected retrospectively from all patients who had the genetic panel for thrombocytopenia between June 2020 and March 2023.

Thrombocytopenia was categorized by severity as mild (100-149 × 10^9^/L), moderate (50-99 × 10^9^/L), or severe (<50 × 10^9^/L) [6]. For the analysis, we chose the nadir platelet count to ensure a consistent and reliable point of comparison.

## 1 Adult Cohort Results

Of 53 patients tested for thrombocytopenia, 39 had a detected genetic variant. Demographics are summarized in Table 1, with details on genetic variants and testing indications provided in Supplementary Table S1. One patient had an incidental finding of a heterozygous pathogenic variant of factor (F)XI with normal FXI levels, but no genetic cause for thrombocytopenia was identified. Another patient with chronic myelomonocytic leukemia underwent genetic testing due to moderate thrombocytopenia. There was no normal historical platelet count prior to chronic myelomonocytic leukemia diagnosis, and the bleeding history was disproportionate to the degree of thrombocytopenia. A *VWF* variant associated with type 2N von Willebrand disease was detected, which may have contributed to the bleeding manifestations.

**Table 1:** Demographics of the adult patients who had positive tests (*n* = 39).

Patient no.	Sex	Clinical phenotype							Platelet count nadir^a^	Gene
		Easy bruising	Epistaxis	HMB	PPH	Bleeding after dental or surgical procedures	Other bleeding symptoms	No significant bleeding or bruising		
1	♀	√		√	√				Moderate	*GP1BA*
2	♂		√						Moderate	*GFI1B*
3	♀						Recurrent hematomas		Moderate	*GP1BB*
4	♀							√	Mild	*CYCS*
5	♀			√		√	Bleeding from minor cuts		Moderate	*TUBB1*
										*TPM4*
6	♀	√		√					Severe	*MYH9*
7	♀		√		√	√			Mild	*FLNA*
8	♂		√						Moderate	*MYH9*
9	♂		√						Severe	*MYH9*
10	♀	√		√	√				Mild	*TUBB1*
11	♂							√	Severe	*WAS*
12	♂							√	Severe	*MYH9*
13	♀				√				Mild	*RUNX1*
										*RUNX1*
14	♀			√					Mild	*TUBB1*
15	♀							√	Severe	*SRC*
16	♀			√					Severe	*ITGA2B*
17	♀			√	√				Mild	*ACTN1*
18	♀			√					Moderate	*TUBB1*
										*TPM4*
19	♂						Oral cavity bleeding		Severe	*GP9*
20	♂							√	Severe	*GFI1B*
21	♂							√	Moderate	*GFI1B*
22	♀					√			Moderate	*MYH9*
23	♀			√					Severe	*MYH9*
24	♂		√						Moderate	*THPO*
25	♀	√		√					Moderate	*VWF*
26	♀							√	Severe	*SRC*
										*VWF*^b^
27	♀	√	√						Severe	*HOXA11*
28	♀		√						Mild	*TUBB1*
29	♀	√							Mild	*TUBB1*
30	♀			√					Moderate	*ACTN1*
31	♂	√							Severe	*MYH9*
32	♀							√	Severe	*FLNA*
33	♀			√					Severe	*MYH9*
34	♀	√		√					Mild	*HPS3*
										*FLI1*
35	♀	√		√					Mild	*GFI1B*
36	♀	√	√	√					Moderate	*HPS3*
										*FLI1*
37	♀		√	√			Oral cavity bleeding		Severe	*MYH9*
38	♀				√	√			Moderate	*VWF*
39	♀				√				Moderate	*F11*^c^

HMB, heavy menstrual bleeding; PPH, postpartum hemorrhage.

a Thrombocytopenia was categorized by severity as mild (100- 149 × 10^9^/L), moderate (50-99 × 10^9^/L), or severe (<50 × 10^9^/L).

b The *VWF* variant was detected incidentally with normal von Willebrand factor (VWF) level and activity and did not account for the thrombocytopenia or the bleeding symptoms.

c The *F11* variant was detected incidentally with normal factor (F)XI levels and did not account for the thrombocytopenia or the bleeding symptoms.

A total of 37 patients (70%) had a genetic variant that could explain their thrombocytopenia, supporting a diagnosis of HT. In 28 of 37 cases (75.6%), testing was prompted after a new diagnosis of thrombocytopenia in a family member, which led to genetic testing of the affected family members.

Notably, a heterozygous pathogenic *SRC* missense variant was identified in 2 thrombocytopenic patients who were investigated for familial myelofibrosis and its known link to HT.

Three patients were classified as having an HT despite having no genetic variant identified. One had moderate thrombocytopenia with skeletal anomalies, another had mild thrombocytopenia with a family history of low platelet count, and third had moderate thrombocytopenia with abnormal platelet aggregometry. This highlights the importance of clinical history and other diagnostic tests for diagnosing inherited bleeding disorders. Further developments are currently underway to expand the range of genetic testing available, such as the National Institute for Health and Care Research – Bleeding, Thrombotic and Platelet Disorders registry [7].

## 2 Pediatric Cohort Results

Of 14 pediatric patients who underwent genetic testing, 8 (57%) had a genetic variant that supported the diagnosis of HT. The mean age was 7.8 ± 2.7 (range: 3-12) years. Demographic data are presented in Table 2, with additional details on genetic findings and testing indications in Supplementary Table S2. Of note, one patient had a *MYH9* variant and a *VWF* variant associated with type 1 von Willebrand disease, which further explained their bleeding symptoms.

**Table 2:** Demographics of pediatric patients who had positive tests (*n* = 8).

Patient number	Sex	Clinical phenotype			Platelet count nadir^a^	Gene
		Easy bruising	Epistaxis	No significant bleeding or bruising		
1	♀			√	Mild	*GFI1B*
2	♂	√	√		Severe	*MYH9*
						*VWF*
3	♂			√	Severe	*MYH9*
4	♀	√			Moderate	*CYCS*
5	♀			√	Moderate	*CYCS*
6	♀			√	Moderate	*MPL*
7	♂			√	Moderate	*GFIB*
						*NBEAL2*
8	♀		√		Moderate	*GP1BB*

a Thrombocytopenia was categorized by severity as mild (100-149 × 10^9^/L), moderate (50-99 × 10^9^/L), or severe (<50 × 10^9^/L).

Although specific treatments are not available for most HTs, accurate diagnosis of patients remains essential. In addition to avoiding unnecessary treatments and interventions, it identifies extrahematopoietic phenotypes, informs appropriate family testing and in some cases, guides the timing of advanced therapies such as stem cell transplantation. The overall detection rate of a genetic variant that could explain the thrombocytopenia in the cohort investigated using the R90 genetic panel was 45 of 67 (67%). This result is slightly higher than a similar study in which the detection rate was 46.6% [8], further validating the diagnostic utility of the R90 panel. Other studies employing different genetic testing methods have reported positive genetic results ranging from 30% to 50% in patients with thrombocytopenia [[9], [10], [11]].

Unlike the study by Gurumurthy et al. [8], which included only patients with platelet counts <100 × 10^9^/L, we expanded our inclusion criteria to patients with platelet counts <150 × 10^9^/L. While individuals with platelet counts between 100 and 150 × 10^9^/L are generally not considered to have a significant hematologic condition and are often not referred to hematologists [12], this range is commonly observed in patients with monogenic, nonsyndromic, mild HTs [13,14]. Mild thrombocytopenia was observed in 11 of 37 (27%) adult and in 1 of 8 (12.5%) pediatric patients who had a variant explaining their thrombocytopenia. This finding reinforces the importance of recognizing cases of mild thrombocytopenia as a potential indicator of HT, which might be overlooked if focusing solely on <100 × 10^9^/L thresholds.

In our cohort, a historical diagnosis of ITP was reported in 56.7% (*n* = 21) of adult and 25% (*n* = 2) of pediatric patients who had thrombocytopenia-related variants. The risk of severe mucosal or spontaneous bleeding in ITP patients with a platelet count > 20 × 10^9^/L is generally low [15]. Therefore, it is crucial to maintain vigilance in cases in which patients present with a bleeding phenotype disproportionate to their platelet count. The misdiagnosis of HTs as ITP can potentially lead to unnecessary interventions, such as splenectomy and immunosuppressive therapies. Such interventions not only fail to resolve the thrombocytopenia but also introduce new risks, including lifelong vulnerability to infections. Additionally, from a clinical perspective, accurate diagnosis of HT is crucial for guiding appropriate management, particularly in the context of perioperative planning.

It is essential to have appropriate testing criteria for molecular investigation to optimize clinical management and appropriate use of resources [16]. In the present study, no statistically significant difference in mean platelet volume (MPV) was observed between patients with detected variants (17.47 ± 4.54) and those without (18.35 ± 4.39) in the adult cohort (*P* = .61). Despite the fact that we could not correlate MPV with the identification of genetic variants, abnormally large platelets were observed on morphology in 11 of 37 (29.7%) adults and 2 of 8 (25%) pediatric patients with thrombocytopenia-related variants. This suggests that the presence of large platelets on blood films may be a more reliable indicator of underlying HT. One limitation of this study is the absence of MPV data for pediatric patients, limiting the ability to perform analysis of this subgroup.

It was noted that 17 of 37 patients (45.9%) with detected variants explaining their thrombocytopenia had severe thrombocytopenia. In the adult cohort, the mean platelet count nadir did not differ significantly between patients with an identified variant (63.84 ± 39.26 × 10^9^/L) and those without (72.50 ± 36.66 × 10^9^/L; *P* = .44). Similar findings were observed in the pediatric cohort, as there was no significant difference in mean platelet nadir in those who had a variant (48.00 ± 54.24 × 10^9^/L) vs those who did not (64.75 ± 24.77 × 10^9^/L; *P* = .35). This result differs from the study by Gurumurthy et al. [8], which suggested patients with a nadir platelet count of <15 × 10^9^/L were significantly less likely to have a genetic variant identified through R90 panel.

Among 37 adults with detected variants supporting the diagnosis of HT, 28 (75.6%) had a family history of thrombocytopenia. In pediatric cases, this was observed in 4 (50%) patients. This finding highlights the importance of considering an underlying genetic etiology when a family history is present. However, the lack of family history should not exclude the consideration of HTs due to the possibility of *de novo* variants, recessive disorders, and/or asymptomatic relatives.

In conclusion, a significant number of patients with detected variants had a family history of thrombocytopenia and/or macrothrombocytopenia in platelet morphology. However, the degree of thrombocytopenia or MPV did not correlate with the detection of a genetic variant. Therefore, we believe clinicians should remain vigilant and consider genetic testing in patients with thrombocytopenia when there is strong clinical suspicion.
