# Supplementary material for: The diagnostic utility of genetic testing in inherited thrombocytopenia: regional multicenter tertiary experience
Source: Res Pract Thromb Haemost. 2025 Apr 24;9(3):102869. doi: 10.1016/j.rpth.2025.102869 (PMC12145818; doi:10.1016/j.rpth.2025.102869)
Supplement: Supplementary Tables S1 and S2 [file mmc1.docx]

**Supplementary Table S1 Reason for testing and genetic results of the adult patients who had positive tests (n= 39)**

| **Patient Number** | **Indication For Testing** | | | | | **Genetic Result** | | | | | |  |
| --- | --- | --- | --- | --- | --- | --- | --- | --- | --- | --- | --- | --- |
|  | **Chronic Thromboc-ytopenia ^(1)^** | **Abnormal Physical Features** | **Macrothrom-bocytopenia** | **Family History of Thromboc-ytopenia** | **Other** | **Gene** | **HGVS Nomenclature** | **Zygosity** | **ACMG Criteria** | **Significance** |  |  |
| 1 |  |  | √ | √ | Confirmation of historic diagnosis ^(2)^ | GP1BA | NM_000173.7: c.413G>T p.(Gly138Val) | Heterozygous | PM2_moderate | VUS |  |  |
| 2 | √ |  |  | √ |  | GFI1B | NM_004188.8: c.503G>T p.(Cys168Phe) | Homozygous | PS3_Supporting, PP1_Strong, PP3_Supporting | Likely pathogenic |  |  |
| 3 |  | √ (DiGeorge syndrome) | √ |  |  | GP1BB | Whole gene deletion | Heterozygous | PVS1_Very strong, PM6_Moderate | Pathogenic |  |  |
| 4 | √ |  |  | √ |  | CYCS | NM_018947.6: c.59C>T p.(Thr20Ile) | Heterozygous | PS4_Supporting, PM2_Moderate, PP1_Strong | Likely pathogenic |  |  |
| 5 | √ |  |  |  |  | TUBB1 | NM_030773.4: c.1219G>A p.(Glu407Lys) | Heterozygous | PM2_Supporting, PP3_Supporting | VUS |  |  |
|  |  |  |  |  |  | TPM4 | NM_001145160.2: c.748G>A p.(Glu250Lys) | Heterozygous | PM2_Supporting, PP3_Supporting | VUS |  |  |
| 6 |  |  | √ | √ |  | MYH9 | NM_002473.6: c.3493C>T p.(Arg1165Cys) | Heterozygous | PS4_Strong, PP4_Strong, PS3_Supporting, PM2_Moderate, PM5_Moderate, PP1_Strong, PP3_Supporting | Pathogenic |  |  |
|  |  |  |  |  |  |  |  |  |  |  |  | |
| 7 | √ |  |  |  |  | FLNA | NM_001110556.2: c.5179G>A p.(Gly1727Ser) | Heterozygous | PM2_Moderate, PP3_Supporting | VUS |  | |
| 8 | √ | √ (Hearing loss) | √ | √ |  | MYH9 | NM_002473.6: c.5521G>A p.(Glu1841Lys) | Heterozygous | PS3_Strong, PS4_moderate, PP4_Supporting, PM2_Moderate, PP2_Supporting, PP3_Supporting | Pathogenic |  | |
| 9 |  |  | √ | √ | Confirmation of historic diagnosis | MYH9 | NM_002473.6: c.5521G>A p.(Glu1841Lys) | Heterozygous | PS3_Strong, PS4_moderate, PP4_Supporting, PM2_Moderate, PP2_Supporting, PP3_Supporting | Pathogenic |  | |
| 10 | √ |  |  |  |  | TUBB1 | NM_030773.4: c.1199G>A p.(Ser400Asn) | Heterozygous | PM2_Supporting, PS4_supporting | VUS |  | |
| 11 | √ |  |  | √ |  | WAS | NM_000377.3: c.256C>T p.(Arg86Cys) | Hemizygous | PS4_Moderate, PM2_Moderate, PM5_Moderate, PP3_Supporting, PP4_Strong | Pathogenic |  | |
| 12 |  |  | √ | √ |  | MYH9 | NM_002473.6: c.5766_5767delinsA p.(Arg1923fs) | Heterozygous | PVS1_Strong, PM2_Moderate | Likely pathogenic |  | |
| 13 | √ |  |  | √ |  | RUNX1 | NM_001754.5: c.1036del p.(Arg346fs) | Heterozygous | PVS1_Strong,  PM2_Moderate | Likely pathogenic |  | |
|  |  |  |  |  |  | RUNX1 | NM_001754.5: c.1102A>T p.(Met368Leu) | Heterozygous | PM2_Moderate | VUS |  | |
| 14 | √ |  |  | √ |  | TUBB1 | NM_030773.4: c.1265A>C p.(Tyr422Ser) | Heterozygous | PP2_Supporting, PP3_Supporting | VUS |  | |
| 15 |  |  |  | √ | Familial myelofibrosis | SRC | NM_198291.3: c.1579G>A p.(Glu527Lys) | Heterozygous | PS4_Moderate, PP1_Strong, PS2_Moderate, PM2_Moderate, PS3_Moderate, PP2_Supporting | Pathogenic |  | |
| 16 | √ |  |  | √ |  | ITGA2B | NM_000419.5: c.668T>G p.(Leu223*) | Heterozygous | PVS1_Very strong, PM2_Moderate | Pathogenic |  | |
| 17 | √ |  |  | √ |  | ACTN1 | NM_001130004.2: c.136C>T p.(Arg46Trp) | Heterozygous | PS4_Moderate, PP1_Strong, PP4_Moderate, PM2_Supporting, PM5_Moderate, PP2_Supporting, PP3_Supporting | Pathogenic |  | |
| 18 | √ |  |  | √ |  | TUBB1 | NM_030773.4: c.1219G>A p.(Glu407Lys) | Heterozygous | PM2_Supporting, PP3_Supporting | VUS |  | |
|  |  |  |  |  |  | TPM4 | NM_001145160.2: c.748G>A p.(Glu250Lys) | Heterozygous | PM2_Supporting, PP3_Supporting | VUS |  | |
| 19 |  |  | √ |  | Confirmation of historic diagnosis ^(2)^ | GP9 | NM_000174.5: c.70T>C p.(Cys24Arg) | Homozygous | PM1_Moderate, PM3_Moderate, PM5_Moderate, PP3_Supporting, PP4_Strong | Pathogenic |  | |
| 20 |  |  | √ | √ |  | GFI1B | NM_004188.8: c.503G>T p.(Cys168Phe) | Heterozygous | PS3_Supporting, PP1_Strong, PP3_Supporting | Likely pathogenic |  | |
|  |  |  |  |  |  |  |  |  |  |  |  | |
| 21 | √ |  | √ | √ |  | GFI1B | NM_004188.8: c.503G>T p.(Cys168Phe) | Heterozygous | PS3_Supporting, PP1_Strong, PP3_Supporting | Likely pathogenic |  | |
| 22 | √ |  |  |  |  | MYH9 | NM_002473.6: c.4562A>G p.(His1521Arg) | Heterozygous | PS4_Moderate, PM2_Moderate, PP1_Supporting, PP3_Supporting, PP4_Strong | Pathogenic |  | |
| 23 | √ |  |  | √ |  | MYH9 | NM_002473.6: c.284C>T p.(Ala95Val) | Heterozygous | PS4_Moderate, PM2_Moderate, PM5_Moderate, PP1_Strong, PP2_Supporting, PP3_Supporting | Pathogenic |  | |
| 24 | √ |  |  | √ |  | THPO | NM_000460.4: c.141+21_*155del (3510bp deletion of exons 4-6) | Heterozygous | PM2_Moderate | VUS |  | |
| 25 | √ |  |  |  |  | VWF | NM_000552.5: c.4010C>T p.(Pro1337Leu) | Heterozygous | PS4_Moderate, PP1_Strong, PM2_Moderate, PS3_Moderate, PP4_Strong | Pathogenic |  | |
|  |  |  |  |  |  | VWF | NM_000552.5: c.2561G>A p.(Arg854Gln) | Heterozygous | PM5_Moderate, PP1_Strong, PP4_Strong | Pathogenic |  | |
| 26 |  |  |  | √ | Familial myelofibrosis | SRC | NM_198291.3: c.1579G>A p.(Glu527Lys) | Heterozygous | PS2_Moderate, PS3_Moderate, PS4_Moderate, PM2_Moderate, PP1_Strong, PP2_Supporting | Pathogenic |  | |
|  |  |  |  |  |  | VWF ^(3)^ | NM_000552.5: c.1730-5C>T | Heterozygous | PP4_supporting | VUS |  | |
| 27 |  | √ |  |  |  | HOXA11 | NM_005523.6: c.248A>G p.(Tyr83Cys) | Heterozygous | PP3_Supporting | VUS |  | |
| 28 | √ |  |  | √ |  | TUBB1 | NM_030773.4: c.264del p.(Asp88fs) | Heterozygous | PVS1_Strong, PM2_Moderate | Likely pathogenic |  | |
| 29 | √ |  |  | √ |  | TUBB1 | NM_030773.4: c.264del p.(Asp88fs) | Heterozygous | PVS1_Strong, PM2_Moderate | Likely pathogenic |  | |
| 30 | √ |  |  | √ |  | ACTN1 | NM_001130004.2: c.136C>T p.(Arg46Trp) | Heterozygous | PS4_Moderate, PP1_Strong, PP4_Moderate, PM2_Moderate, PM5_Moderate, PP2_Supporting, PP3_Supporting | Pathogenic |  | |
| 31 | √ |  |  | √ |  | MYH9 | NM_002473.6: c.3493C>T p.(Arg1165Cys) | Heterozygous | PS4_Strong, PP4_Strong, PS3_Supporting, PM2_Moderate, PM5_Moderate, PP1_Strong, PP3_Supporting | Pathogenic |  | |
| 32 | √ |  |  |  |  | FLNA | NM_001110556.2: c.2492_2497dup p.(Asn831_Asp832dup) | Heterozygous | PM2_Moderate | VUS |  | |
| 33 | √ |  |  | √ |  | MYH9 | NM_002473.6: c.4337T>C p.(Phe1446Ser) | Heterozygous | PS4_Moderate, PM2_Moderate, PM5_Supporting, PP2_Supporting, PP3_Supporting | Likely pathogenic |  | |
| 34 | √ |  |  | √ |  | HPS3 | NM_032383.5: c.1379_1381del p.(Arg460_Gln461delinsLys) | Heterozygous | PM2_Moderate, PM4_supporting | VUS |  | |
|  |  |  |  |  |  | HPS3 | NM_032383.5: c.1870G>T p.(Glu624*) | Heterozygous | PVS1_Very strong, PM2_Moderate, PM3_supporting | Pathogenic |  | |
|  |  |  |  |  |  | FLI1 | NM_002017.5: c.369_370del p.(Arg123fs) | Heterozygous | PVS1_Very strong, PM2_Moderate | Pathogenic |  | |
| 35 | √ |  | √ | √ |  | GFI1B | NM_004188.8: c.503G>T p.(Cys168Phe) | Heterozygous | PS3_Supporting, PP1_Strong, PP3_Supporting | Likely pathogenic |  | |
| 36 |  |  |  | √ |  | HPS3 | NM_032383.5: c.1379_1381del  p.(Arg460_Gln461delinsLys) | Heterozygous | PM2_Moderate, PM4_supporting | VUS |  | |
|  |  |  |  |  |  | HPS3 | NM_032383.5: c.1870G>T p.(Glu624*) | Heterozygous | PVS1_Very strong, PM2_Moderate, PM3_supporting | Pathogenic |  | |
|  |  |  |  |  |  | FLI1 | NM_002017.5: c.369_370del p.(Arg123fs) | Heterozygous | PVS1_Very strong, PM2_Moderate | Pathogenic |  | |
| 37 |  |  | √ | √ |  | MYH9 | NM_002473.6: c.5766_5767delinsA p.(Arg1923fs) | Heterozygous | PVS1_Strong, PS4_Moderate, PM2_Moderate | Likely pathogenic |  | |
| 38 | √ | √ |  |  | Bleeding symptoms unexplained by the platelet count | VWF | NM_000552.5: c.2561G>A p.(Arg854Gln) | Homozygous | PM5_Moderate, PP1_Strong, PP4_Strong | Pathogenic |  | |
| 39 | √ |  |  |  |  | F11 ^(4)^ | NM_000128.4:c.976C>T p.(Arg326Cys) | Heterozygous | PS4_Moderate, PM5_Supporting, PP3_Supporting, PP4_Strong | Likely pathogenic |  | |

ACMG: American College of Medical Genetics and Genomics, HGVS: Human Genome Variation society, VUS: variant of uncertain significance, ^(1)^ lasting more than one year and a platelet count of <150×10⁹/L with no historic normal platelet counts including thrombocytopenia unresponsive to standard treatments for acquired thrombocytopenia (ITP), ^(2)^ Including abnormal platelet function testing, ^(3)^ The VWF variant was detected incidentally with normal vWF level and activity and did not account for the thrombocytopenia or the bleeding symptoms,^(4)^ The F11 variant was detected incidentally with normal FXI levels and did not account for the thrombocytopenia or the bleeding symptoms

**Supplementary Table S2 Reason for testing and genetic results of the paediatric patients who had positive tests (n= 8)**

| **Patient Number** | **Indication For Testing** | | | | | **Genetic Result** | | | | |
| --- | --- | --- | --- | --- | --- | --- | --- | --- | --- | --- |
|  | **Chronic Thrombocy-teopenia (2)** | **Abnormal Physical Features** | **Macrothromb-ocytopenia** | **Family History of Thrombocytopenia** | **Other** | **Gene** | **HGVS Nomenclature** | **Zygosity** | **ACMG Criteria** | **Significance** |
| 1 | √ |  | √ | √ |  | GFI1B | NM_004188.8: c.503G>T p.(Cys168Phe) | Heterozygous | PS3_Supporting, PP1_Strong, PP3_Supporting | Likely pathogenic |
| 2 | √ |  |  | √ |  | MYH9 | NM_002473.6: c.4270G>A p.(Asp1424Asn) | Heterozygous | PS3_Strong, PS4_Moderate, PM2_Moderate, PM5_Moderate, PP1_Strong, PP2_Supporting, PP3_Supporting, PP4_Moderate | Pathogenic |
|  |  |  |  |  |  | VWF | NM_000552.5: c.4751A>G p.(Ty1584Cys) | Heterozygous | N/A | Established risk allele ^*^ |
| 3 | √ |  |  |  |  | MYH9 | NM_002473.6: c.4270G>A p.(Asp1424Asn) | Heterozygous | PS3_Strong, PS4_Moderate, PM2_Moderate, PM5_Moderate, PP1_Strong, PP2_Supporting, PP3_Supporting, PP4_Moderate | Pathogenic |
| 4 | √ |  |  | √ |  | CYCS | NM_018947.6: c.59C>T p.(Thr20lle) | Heterozygous | PS4_Supporting, PM2_Moderate, PP1_Strong | Likely pathogenic |
| 5 | √ |  |  |  |  | CYCS | NM_018947.6: c.154G>A p.(Ala52Thr) | Heterozygous | PS4_Moderate, PM1_Supporting, PM2_Moderate, PM5_Supporting | Likely pathogenic |
| 6 | √ |  |  |  |  | MPL | NM_005373.3: c.378del p.(Phe126fs) | Heterozygous | PVS1_Very Strong, PM2_Supporting, PM3_Strong | Pathogenic |
| 7 | √ |  | √ | √ | Confirmation of historic diagnosis ^(2)^ | GFIB | NM_004188.8: c.503G>T p.(Cys168Phe) | Heterozygous | PS3_Supporting, PP1_Strong, PP3_Supporting | Likely pathogenic |
|  |  |  |  |  |  | NBEAL2 | NM_015175.3: c.8164-1G>A | Homozygous | PVS1_Moderate, PM2_Moderate | VUS |
| 8 | √ | √ (DiGeorge syndrome) |  |  |  | GP1BB | Whole gene deletion | Heterozygous | PVS1_Very strong, PM6_Moderate | Pathogenic |

ACMG: American College of Medical Genetics and Genomics, HGVS: Human Genome Variation society, VUS: variant of uncertain significance, ^(1)^ lasting more than one year and a platelet count of <150×10⁹/L with no historic normal platelet counts including thrombocytopenia unresponsive to standard treatments for acquired thrombocytopenia (ITP), ^(2)^ Including abnormal platelet function testing,

^*^Schmidt RJ, Steeves M, Bayrak-Toydemir P, Benson KA, Coe BP, Conlin LK, et al. Recommendations for risk allele evidence curation, classification, and reporting from the ClinGen Low Penetrance/Risk Allele Working Group. Genet Med. 2024;26(3):101036. doi: 10.1016/j.gim.2023.101036.
